# The Department of Modern Nursing

**Published:** 1914-08-08

**Authors:** 


### THE DEPARTMENT OF MODERN NURSING.*

### North London or University College Hospital.

Between the years 1897 and 1905 this hospital was entirely rebuilt and now presents within the somewhat cramped limits of its site an admirably planned collection of buildings. The ingenuity with which every inch of space has been utilised is specially observable in the nurses' quarters. An exhilarating effect of air, light, and space has been produced. The hospital has its own Medical School and Students' House. It serves a large and poor neighbourhood, and has extensive out-patient and casualty departments. By means of five different Samaritan Funds it assists indigent patients and relieves its own beds by discharging to convalescent homes those who are fit to be moved as soon as possible. The Schiff House of Recovery is very valuable in this connection. The continual succession of acute cases tends to keep the work of the hospital, including the nursing, at high pressure. The number of beds is 305 with a daily average of occupied beds in 1912 of 279.7. The nursing staff numbers 121, including matron, home sister, and night superintendent; two district nurses, one masseuse, two *x*-ray nurses, fifteen sisters, forty-eight nurses, and fifty probationers.

### The Supply of Probationers.

The training school is fortunate in being able to attract a high class of probationers, and the waiting list is always a long one. This results, it may be surmised, in great measure from the close connection between the hospital and University College. The professors, the members of the medical staff, and their wives take a personal interest in the hospital, and the bonds are drawn closer by the existence of an enthusiastic and admirably managed ladies' league. The advantages which the training school has to offer to probationers of good previous education are thus brought under the notice of a wide circle able to feed the supply. Were the same system of cordiality to friends of the hospital followed in institutions which at present are continually deploring the lack of good candidates it is probable that an equally good result could be obtained. Candidates are considered to be on probation for six months after entering the hospital. It is found that about one in ten proves unsuitable from one cause or another, though the proportion of those who fail naturally varies a good deal. The age limit is 23-30. Salaries are £8, £12, and £16; nurses, £24 to £27; sisters, £35 to £50.

### The Probationer's Course.

Lectures are given once a week by members of the staff on anatomy and physiology, and are supplemented by class instruction with the home sister. These take place during duty hours. The examinations are held in April and include both practical and theoretical work. The ward scheme provides for probationers remaining three months at a time in each ward to which they may be assigned. After the first year probationers are appointed junior staff nurses, and in their third year they become senior staffs. The fine cruciform wards containing 24 beds, and in the case of the children's ward 28 cots, are under a sister, two staff nurses, and two probationers. They are exceptionally handsome and commodious and much thought has been expended in utilising the space to the utmost for the comfort of patients and the convenience of the nursing. The plan on which they are built enables the patients who are dying or acutely ill to be removed from the bustle of the ward to the far end. Such facilities for studying the individual requirements of patients undoubtedly aid in developing the nurse's sympathies and enlarging her ideas of responsibility towards the sick. It is the dead level of the ordinary ward, excluding perforce any kindly consideration for personal idiosyncrasies, which tends to make nurses into machines while they are yet in training.

### Theatre and Out-Patient Work.

Seven nurses are always employed under the Sister in the theatres, and six months' experience in theatre work is the rule, although there are some nurses who do not take it. The out-patient department requires eight nurses under the Sister, and considerable experience in minor surgery is thus obtained. There is a good light department, two nurses being always engaged in *x*-ray work.

### The Fourth Year.

After completing their three years' course of training and passing their final examination members of the staff are eligible to serve on the private staff or for appointments in the hospital, where valuable experience in housekeeping and other administrative posts may be gained. The hospital is a recognised training centre under the Central Midwives Board, and its own nurses are admitted to this branch of training at a reduced fee of £21 for the course. It is carried on both in the district and in the maternity wards of the hospital.

### Private Nursing Institute.

The Trained Nurses' Institution in connection with the hospital is in Huntley Street, Gower Street, W.C. It numbers from thirty-five to forty nurses, who work after the expiration of their fourth year's service on the co-operative system, paying $7\frac{1}{2}$ per cent. commission to the institute on their cases and a fixed sum for board and lodging when not at a case.

* Previous articles of this series appeared in The Hospital of May 16 and 30, June 13, July 11 and 25.

The Nurses' Life in the Home.

The nurses' quarters are admirably planned and very comfortable. Each member of the staff has a cheerful bed-sitting-room for her own use, and the community rooms are all good. Papers and periodicals are supplied from a special fund. Probationers have two hours off duty every day, with four hours twice a week, and an off day once a month. They are entitled to three weeks' holiday in the year and the seniors to a month. The sickness rate is unusually low. The excellent standard of physical fitness which the Matron is able to maintain among the nurses is due no doubt partly to the bright and airy quarters. But absence of worry and a well-planned time-table counts higher than even pleasant surroundings in enabling nurses to support fatigue without undue strain. The commissariat is good and varied.

Cost of the Nursing Department.

The accounts of the nursing department are kept entirely distinct from those of the hospital, and the average cost of the nursing staff per bed in the year 1911 under the heads of laundry, maintenance, and salaries was £16.5, the lowest average among London clinical hospitals. The average cost of the nursing per head was £38.7. The average cost of the nurses' board works out at £18.2 a year, or 6s. 11½d. per head per week.

